# Evaluation of Molecular Properties versus In Vivo Performance of Aflibercept, Brolucizumab, and Ranibizumab in a Retinal Vascular Hyperpermeability Model

**DOI:** 10.1167/tvst.11.10.36

**Published:** 2022-10-25

**Authors:** William Schubert, Carsten Terjung, Ashique Rafique, Carmelo Romano, Philipp Ellinger, Kay D. Rittenhouse

**Affiliations:** 1 Former employee, Bayer AG, Wuppertal, Germany; 2 Bayer AG, Wuppertal, Germany; 3 Regeneron Pharmaceuticals, Inc., Tarrytown, NY, USA; 4 Bayer Consumer Care AG, Basel, Switzerland

**Keywords:** preclinical, anti-VEGFs, durability, potency, computational modeling

## Abstract

**Purpose:**

To evaluate the molecular, pharmacokinetic, and pharmacological properties of three anti-vascular endothelial growth factor (VEGF) agents—aflibercept, brolucizumab, and ranibizumab—and to provide a prediction of the optimal design of an intravitreal VEGF challenge in rabbits to assess the preclinical in vivo activity of the different anti-VEGF agents.

**Methods:**

Biochemical analyses and cellular and animal models of retinopathy were used to characterize anti-VEGF efficacy. Anti-VEGF biochemical binding affinity was determined through a kinetic exclusion assay. The in vitro potency was investigated by a calcium mobilization assay. Pharmacokinetic parameters were estimated for each drug to predict intraocular exposure relationships among the agents. The in silico modeling efforts informed the design of an in vivo rabbit model of VEGF-induced retinal hyperpermeability to determine the extent of VEGF neutralization in vivo. Consequently, data generated from the in vivo study enabled pharmacokinetic analysis and the generation of a logistical model describing the impact of the anti-VEGF agents on the VEGF-induced vascular leakage in rabbits.

**Results:**

The three anti-VEGF agents ranked from most efficacious to least efficacious as aflibercept, brolucizumab, and ranibizumab, with results consistent and significant within each individual characterization experiment.

**Conclusions:**

This composite study demonstrated how the molecular properties of aflibercept, brolucizumab, and ranibizumab translate into differences of in vivo efficacy, with results in line with the reported literature.

**Translational Relevance:**

In silico, in vitro, and in vivo integrated studies provide information that enables the enhanced characterization of translational properties of anti-VEGF agents currently used for the treatment of retinal diseases.

## Introduction

Treatment for retinal diseases, including neovascular age-related macular degeneration, includes anti-vascular endothelial growth factor (VEGF) injections, which attenuate the capacity of VEGF to bind to VEGF receptors.

Anti-VEGF therapies have demonstrated medically important and impactful improvements in vision for the patients receiving these treatments. Accordingly, in the years since the approval of the first anti-VEGFs for the treatment of neovascular age-related macular degeneration (pegaptanib and ranibizumab),^1^^–^^3^ multiple anti-VEGF therapies have been developed and approved for use. As described in the prescribing guidelines, aflibercept is a recombinant fusion protein that consists of the extracellular domains of human VEGF receptors 1 and 2 fused to the fragment crystallizable (Fc) portion of human immunoglobulin (Ig) G1. Aflibercept binds VEGF-A, VEGF-B, and placental growth factor, sequestering these molecules and thus decreasing signaling by VEGF receptors.^4^^,^^5^ Brolucizumab is a humanized monoclonal single-chain variable domain antibody fragment that binds VEGF-A,^6^ and ranibizumab is a humanized monoclonal IgG1κ isotype fragment (lacking an Fc region) that binds VEGF-A.^1^

The effectiveness of intravitreal anti-VEGF therapies—that is, robust improvements in visual and anatomic outcomes and the subsequent maintenance of these improvements—may be contingent on both individual drug characteristics and appropriate posology. Several studies have investigated the molecular properties of macromolecules and preclinical and clinical characteristics.^7^^–^^10^

In animal experiments, the study design should always aim at striking a balance between providing meaningful results and limiting the number of animals used. For VEGF-induced vascular leakage in rabbits, this goal is particularly challenging, because the desired anti-VEGF efficacy changes over time, and only limited time points can be assessed in each individual due to increasing resistance to VEGF after multiple injections or when too short intervals between intravitreal VEGF injections are chosen. Different data sources were combined to provide a study design that did not require the repetition and pooling of studies,^11^ but still provided a meaningful discrimination of the investigated anti-VEGF agents. The aim of this study was to evaluate the relationships between the molecular properties, ocular pharmacokinetics, and the pharmacology of the anti-VEGF agents aflibercept, brolucizumab, and ranibizumab, and to apply a framework of mathematical models to characterize their efficacy in a preclinical animal model of retinopathy. This efficacy is demonstrated by cell-based studies and the in vivo reduction and maintenance of decreased or absent retinal vascular leakage.

## Methods

### Biochemical Binding Assays for Aflibercept, Brolucizumab, and Ranibizumab

The rank order of binding affinity strengths of commercially available aflibercept (Regeneron, Inc., Tarrytown, NY), brolucizumab (Novartis Pharma, Basel, Switzerland), and ranibizumab (Genentech, Inc., South San Francisco, CA) to recombinant human VEGF-A165 was determined by a kinetic exclusion assay (KinExA) with a KinExA 3200, including autosampler (Sapidyne Instruments, Boise, ID). The equilibrium curve was generated by titrating VEGF-A165 while keeping the concentration of the VEGF inhibitor constant. Concentrations of aflibercept, brolucizumab, and ranibizumab were chosen as described by Papadopoulos et al.,^12^ Rodrigues et al.,^13^ and Szabó et al.^14^

Human VEGF-A165 (50 µg) was immobilized onto 75 mg azlactone beads (ThermoFisher Scientific, Waltham, MA), suspended in 1.5 mL of phosphate-buffered saline (PBS), and rotated at 4°C overnight. After removing the supernatant, the beads were incubated for 1 hour at room temperature in 1.0 mL PBS with 10 mg/mL bovine serum albumin (BSA). The beads were washed three times with PBS, resuspended in 30 mL of PBS, and used immediately.

Inhibitor-VEGF mixtures were equilibrated at room temperature (23°C) for 5 to 120 hours, using a PBS buffer containing 0.01% by volume Tween-20, 0.02% NaN_3_, and 1 mg/mL BSA (at a pH of 7.4).

The use of binding curves at two or more different concentrations of constant binding partners allows the software to fit the binding parameters more precisely. Co-complex mixtures per anti-VEGF contained aflibercept (constant concentration range of 2.5, 5.0, and 50.0 pM) with VEGF-A165 (titration concentration range 48.8 fM–1 nM), brolucizumab (constant concentration range 25, 50, and 400 pM) with VEGF-A165 (titration concentration range 0.12 pM–4 nM) or ranibizumab (constant concentration range 50–400 pM) with VEGF-A165 (titration concentration range 1.46 pM–10 nM). All experiments were carried out in duplicate.

Equilibrated mixtures were injected between 2 and 30 minutes through a column of VEGF-A165-coupled microbeads in the KinExA system at a flow rate of 0.25 mL/min. Bead contact time was 0.5 seconds, permitting unbound VEGF inhibitors to be captured by the beads without perturbing the equilibrium state of the solution. Captured VEGF inhibitors were quantified with Alexa 647-conjugated Protein-L (GenScript, Piscataway, NJ), goat polyclonal antihuman (Fc) IgG, or anti-human F(ab’)2 fragment specific for light-chain antibodies (Jackson ImmunoResearch Laboratories, West Grove, PA).

The equilibrium dissociation constant (*K_D_*) was obtained from nonlinear regression analysis of the data with a one-site homogeneous binding model. The “drift correction” option was used where appropriate. Binding data were analyzed globally using the N-curve feature in the KinExA Pro software (version 4.3.11). The *K_D_* 95% confidence interval was calculated by fitting the data points to a theoretical *K_D_* curve, as described in the KinExA protocol. The 95% confidence interval is given as *K_D_* low and *K_D_* high, as described by Darling and Brault.^15^

### In Vitro Calcium Mobilization Assay

Human umbilical vein endothelial cells (Lifeline Cell Technology, Carlsbad, CA) were seeded in 96-well plates (black, with a transparent bottom) at a density of 1.5 × 10^4^ cells/well and cultured for 24 hours in an incubator at 37°C (5% CO_2_). The culture medium was then carefully removed and replaced with a premixed loading buffer, of which 50 mL contained 0.15 mL 10% (v/v) Pluronic, 0.1 mL dimethyl sulfoxide, 5 mg Fluo-8 AM (Abcam, Cambridge, MA), Tyrode's solution (calcium-free Tyrode buffer [Pan Biotech, Aidenbach, Germany] with 2 mM Ca) 49.25 mL + 0.5 mL Brilliant Black BN (20 mg/mL), and 0.25 mL probenecid (0.5 M in 0.5 N NaOH).

The cells were then incubated for 55 minutes at 37°C (5% CO_2_). Human VEGF-A165 and the antibody mix were preincubated for 5 minutes before addition to the wells. Direct kinetic measurement of calcium mobilization was performed immediately at an excitation wavelength of 490 nm and an emission wavelength of 525 nm.

### Optimization of In Vivo Rabbit Study Designs by In Silico Modeling

The pharmacokinetics of VEGF and the anti-VEGF agents after intravitreal injection were calculated using a minimal physiology-based model, based on the assumed predominant elimination of biologics by the anterior route, via diffusion through the vitreous and elimination from the anterior chamber through turnover of aqueous humor.^16^^,^^17^ Ocular elimination half-life estimates were calculated considering the geometry of the rabbit eye and the molecular weight-based hydrodynamic radii of the VEGF protein, the respective anti-VEGF agents, and the VEGF/anti-VEGF complexes using the modeling framework established by Hutton-Smith et al.^17^ Initial estimates for the binding affinity of the anti-VEGF agents were taken from literature references,^18^ with correction for body temperature and binding ratio between anti-VEGF agents and VEGF. The pharmacological effect was predicted based on a semi-mechanistic model of VEGF-induced hyperpermeability in rabbits.^19^ Supplementary Tables S1 and S2 summarize the selected input parameters of the initial model. The outcomes of the in silico modeling were used to design the in vivo rabbit VEGF-A165–induced retinal vascular permeability study using aflibercept, brolucizumab, and ranibizumab. More information on how the model was developed is available in the Supplementary Information.

### In Vivo Rabbit Experimental Model

A human VEGF-A165–induced rabbit retinal vascular hyperpermeability model was used to estimate the inhibition of leakage and duration of activity for rabbit eye-scaled clinical doses of aflibercept, brolucizumab, and ranibizumab. These in vivo rabbit experiments were conducted according to Directive 2010/63/UE of the European Convention for the Protection of Vertebrate Animals used for Experimental and Other Scientific Purposes, and the Association for Research in Vision and Ophthalmology Statement for the Use of Animals in Ophthalmic and Vision Research.

**Figure 1.**
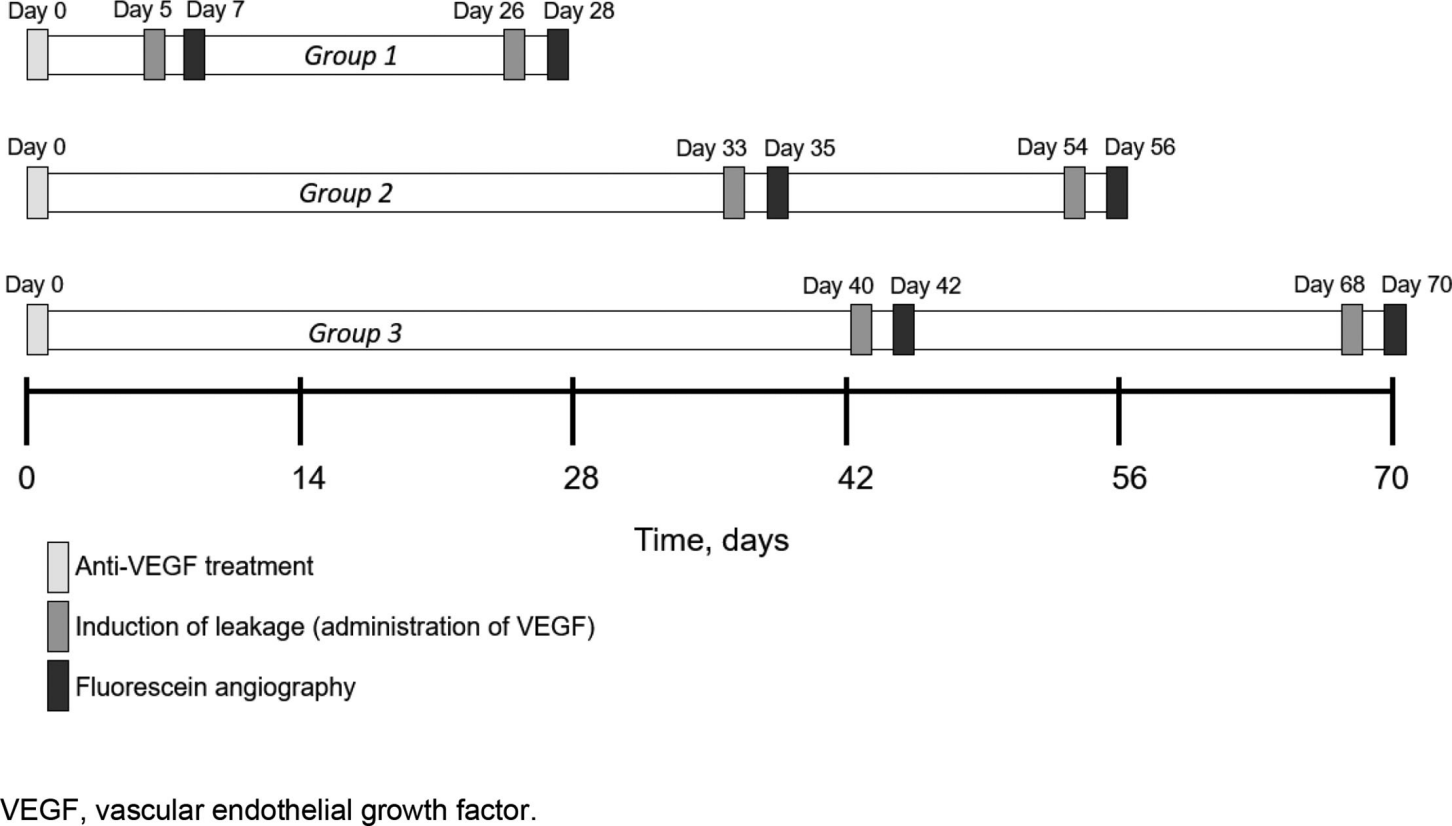
Study design of the in vivo rabbit experimental model.

After quarantine and acclimation, 176 Dutch belted rabbits (Iris-Pharma, La Gaude, France) were randomized into study groups based on body weight measured at baseline. Study groups contained seven animals per agent per time point. Time points for evaluations are described in Figure 1.

Masked clinical doses of each anti-VEGF were scaled to rabbit eyes (25 μL injection of each agent) and resulted in approximately 50% of the clinical dose for each: aflibercept 1.00 mg, brolucizumab 3.00 mg, and ranibizumab 0.25 mg. At baseline, the anti-VEGF injections were administered in the vitreous fluid of the rabbit's right eye (Fig. 1). Satellite groups of rabbits were administered the anti-VEGF treatment in both eyes, in tandem with the respective dosing schedule for experimental animals. These satellite animals were used for the collection of additional pharmacokinetic data points for the noncompartmental analysis of anti-VEGF exposure.

Based on the in silico modeling, induction of leakage was produced via 50 µL intravitreal injection of 500 ng recombinant human VEGF-A165 at day 5 (1 week), day 26 (4 weeks), day 33 (5 weeks), day 40 (6 weeks), day 54 (8 weeks), and day 68 (10 weeks). At 47 ± 3 hours after induction, sodium fluorescein (a concentration of 10% sodium fluorescein in 0.9% saline solution, at a dose of 50 mg/kg) was injected via the marginal ear vein in anti-VEGF–treated animals. At approximately 30 seconds, 1 minute, and 2 minutes after intravenous fluorescein tracer injection, a retinal angiography assessment was performed to observe the vascularization using a Heidelberg retinal angiograph in the right eye. Animals were euthanized after the second fluorescein angiography assessment, and ocular tissues were collected for pharmacokinetic analysis.

### Bioanalytics and Noncompartmental Pharmacokinetic Analysis

Vitreous samples were collected from both healthy satellite animals and animals that had received their second and final VEGF injection after the leakage assessment. Total concentrations of aflibercept, brolucizumab, and ranibizumab in vitreous humor were determined via enzyme-linked immunosorbent assay.

Aflibercept concentrations were determined from rabbit vitreous after dilution with buffer on a Gyrolab xP workstation using the Gyrolab generic pharmacokinetics kit according to the manufacturer's instructions (Uppsala, Sweden). This kit included sample buffers, a Bioaffy 1000 high-capacity compact disc, and labeled ready-to-use capture and detection reagents. The capture reagent was a biotinylated llama antibody fragment (affinity ligand) that specifically binds to the Fc fragment of all four human IgG subclasses without cross-binding to animal IgG. The detection reagent was an anti-human IgG antibody labeled with Alexa Fluor-647. Rabbit vitreous samples (blinded to the investigator) were analyzed concurrently with calibration samples freshly prepared from 1% BSA in PBS Tween buffer, as well as frozen quality control (QC) samples prepared from blank rabbit vitreous matrix that were treated in the same way as the study samples. Before analysis, calibration, QC, and study samples were diluted with the Gyrolab pharmacokinetics kit sample dilution buffer (reagent F), resulting in a minimum required dilution of 10-fold before being loaded onto the compact disc. Data acquisition at the 1% photomultiplier tube level was used. Regression was performed by Gyrolab Evaluator with a nonweighted five-parameter logistic regression algorithm. The working range of the method was from 6.25 ng/mL (lower limit of quantification) to 30,000 ng/mL. Vitreous samples with concentrations exceeding 30,000 ng/mL were diluted with blank rabbit vitreous before analysis.

Ranibizumab concentrations were determined from rabbit vitreous after dilution with buffer on a Gyrolab xP workstation with fluorescence readout, using an immobilized biotinylated goat anti-human IgG antibody (SouthernBiotech; Cat. No. 2049-08) as capture molecule and an Alexa Fluor 647-labeled goat anti-human IgG antibody (Jackson ImmunoResearch Laboratories; lot number 150540) as the detection reagent. Vitreous samples were analyzed concurrently with freshly prepared calibration samples and frozen QC samples prepared from blank rabbit vitreous matrix. QC samples were treated in the same way as the study samples. Before analysis, calibration, QC, and study samples were diluted with buffer (Gyrolab Rexxip HX buffer), resulting in a minimum required dilution of 20-fold before being loaded onto the compact disc. Data acquisition at the 1% photomultiplier tube level was used. Regression was performed by Gyrolab Evaluator with a nonweighted five-parameter logistic regression algorithm. The working range of the method was from 3.0 ng/mL (lower limit of quantification) to 8000 ng/mL. Vitreous samples with concentrations exceeding 8000 ng/mL were diluted with blank rabbit vitreous before analysis.

Quantification of brolucizumab from rabbit vitreous was conducted by means of a target capture enzyme-linked immunosorbent assay in a 96-well format on a meso scale discovery platform using electrochemiluminescence detection. Briefly, meso scale discovery standard plates were coated with biotinylated recombinant human VEGF target as the capture reagent (R&D Systems, Minneapolis, MN; lot number II6319081), followed by blocking with PBS (pH 7.5) containing 3% BSA. Vitreous samples were diluted with PBS/Tween-20 (pH 7.5) containing 0.25% BSA, resulting in a minimum required dilution of 50-fold, before being loaded onto the plate. Detection was then conducted using ruthenium-labeled protein-L (Pierce Recombinant Protein-L, REF 21189, lot number UA279730; ThermoFisher Scientific). Vitreous samples were analyzed concurrently with freshly prepared calibration samples and frozen QC samples were prepared from blank rabbit vitreous matrix. QC samples were treated in the same way as the study samples. Regression was performed with a 1/(*y*^2^)-weighted five-parameter logistic regression algorithm. The working range of the method was from 20.0 ng/mL (lower limit of quantification) to 1000 ng/mL (vitreous samples with concentrations exceeding this were diluted with PBS buffer before analysis). The dilution procedure was validated using dilution QC samples treated in the same way as the study samples.

The concentration–time profile data for each respective anti-VEGF agent treatment group were pooled and evaluated using noncompartmental analysis (pharmacokinetics software package Phoenix 8.0; Certara, Princeton, NJ).

### Pharmacokinetic and Pharmacodynamic Evaluation of Rabbit In Vivo Study Results

A post hoc comparison of the predicted vitreous concentration–time profiles for anti-VEGF agents with actual total concentrations measured by a ligand-binding assay was performed. Furthermore, leakage scores, which were assessed by the extent of fluorescein visualized in the optical coherence tomography fluorescein angiography imaging (Supplementary Fig. S1), were categorized into two groups: no to mild leakage (score of <2) and moderate to severe leakage (score of ≥2); these categories were assigned a probability of 0 or 1 for each individual leakage assessment. Vehicle data at week 10 were excluded from further evaluation owing to unusually low leakage scores at this observation time point. Based on the observed pharmacokinetics of the anti-VEGF agents and their in vitro affinity binding data, the concentration of free VEGF (i.e., not bound by the administered anti-VEGF agents) in the vitreous humor was predicted by the in silico model. The model-predicted area under the concentration–time curve of free exogenous VEGF in the vitreous between VEGF injection and leakage observation was normalized to the vehicle group (100% free VEGF). This was used as the basis for fitting a logistical model to describe the impact of anti-VEGF intraocular pharmacokinetics on the amount of vitreous free VEGF over time and the resulting probability of moderate to severe retinal leakage in the treated rabbits.

## Results

### Biochemical Binding Assays for Aflibercept, Brolucizumab, and Ranibizumab

**Figure 2.**
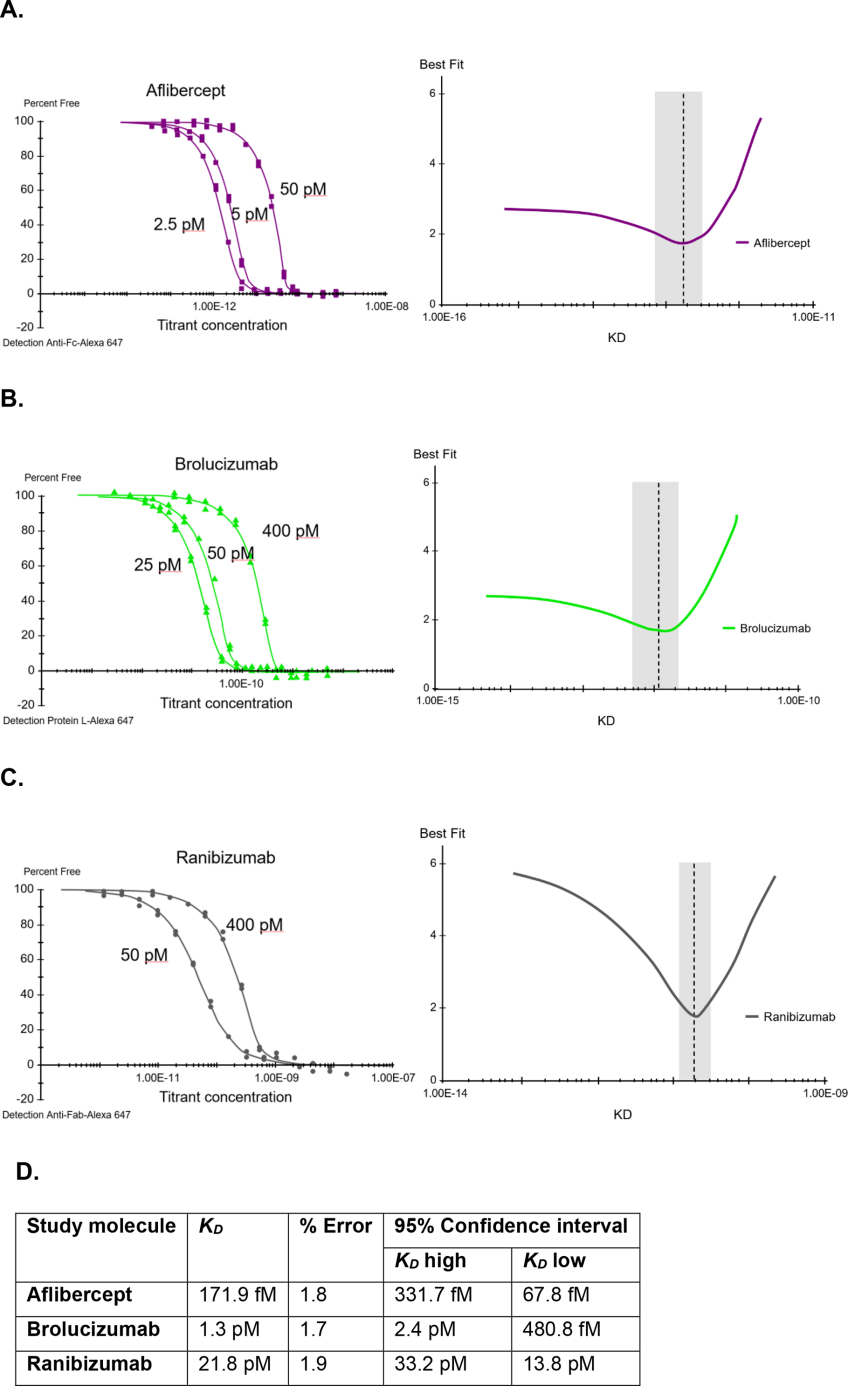
Binding affinity for aflibercept, brolucizumab, and ranibizumab binding to human VEGF-A165 ligand. Solution-based equilibrium binding analysis of (A) aflibercept binding to VEGF-A165, (B) brolucizumab binding to VEGF-A165, (C) ranibizumab binding to VEGF-A165, and (D) binding affinity summary table.

The solution affinity assay quantified the *K_D_* values for the commercially available anti-VEGFs (Fig. 2). The *K_D_* for aflibercept was 171.9 fM (range, 67.8–331.7 fM), for brolucizumab was 1.3 pM (range, 480.8 fM–2.4 pM), and for ranibizumab was 21.8 pM (range, 13.8–33.2 pM). Accordingly, rank order from highest to lowest binding affinity (lowest *K_D_* = highest binding affinity) was aflibercept, brolucizumab, and ranibizumab, with nonoverlapping 95% CIs around the mean *K_D_* for each concentration.

### In Vitro Calcium Mobilization Assay

**Figure 3.**
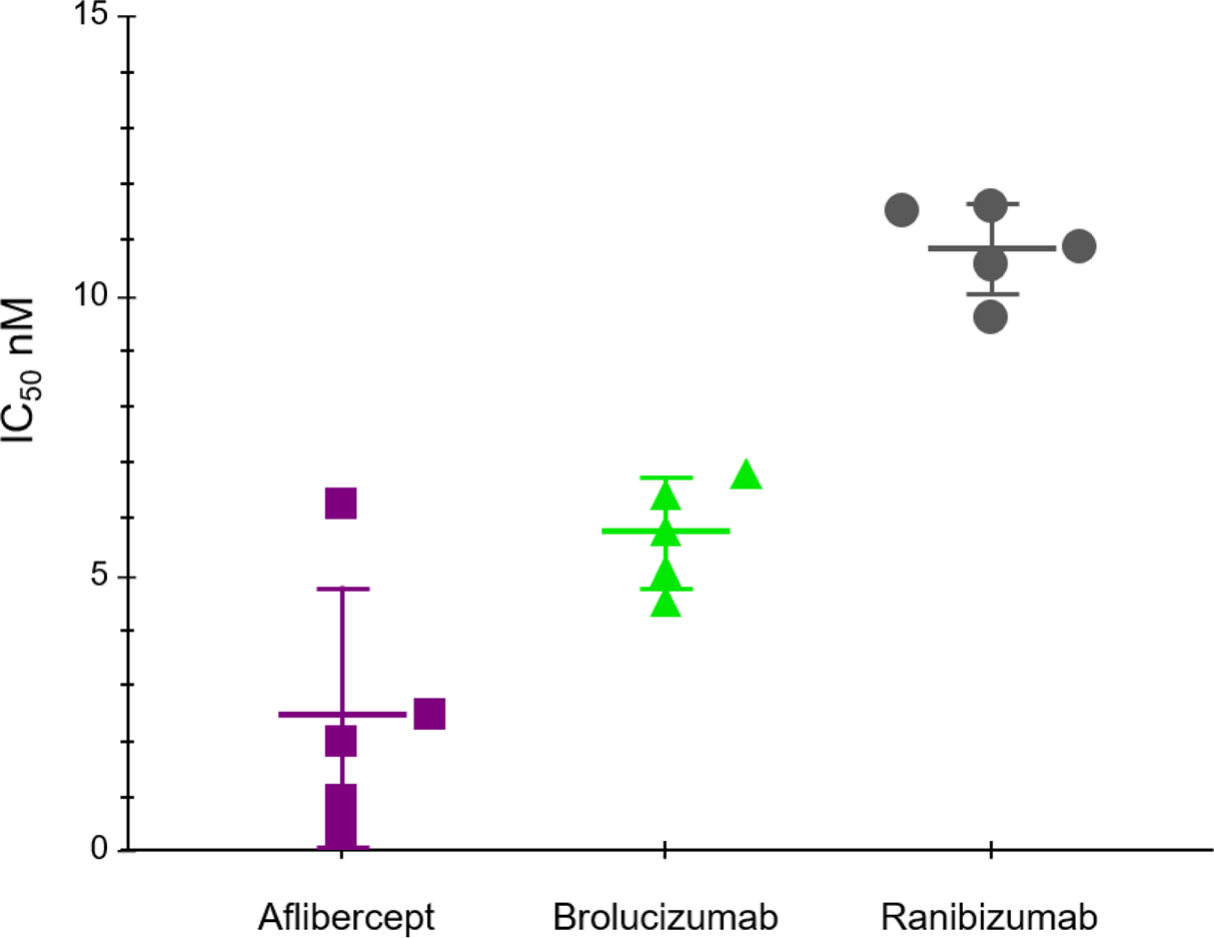
Calcium mobilization in HUVECs after VEGF stimulation.

The rank order observed between study molecules in the in vitro human umbilical vein endothelial cell calcium mobilization assay (Fig. 3) was consistent with the biochemical binding assay data. The half-maximal inhibitory concentration value was 2.42 nM for aflibercept, 5.74 nM for brolucizumab, and 10.82 nM for ranibizumab. Using Tukey's multiple comparisons test, aflibercept was significantly more potent than brolucizumab and ranibizumab (aflibercept vs. brolucizumab, *P* = 0.0126; aflibercept vs. ranibizumab, *P* < 0.0001; brolucizumab vs. ranibizumab, *P* = 0.0005).

### In Vivo Rabbit Experimental Model

**Figure 4.**
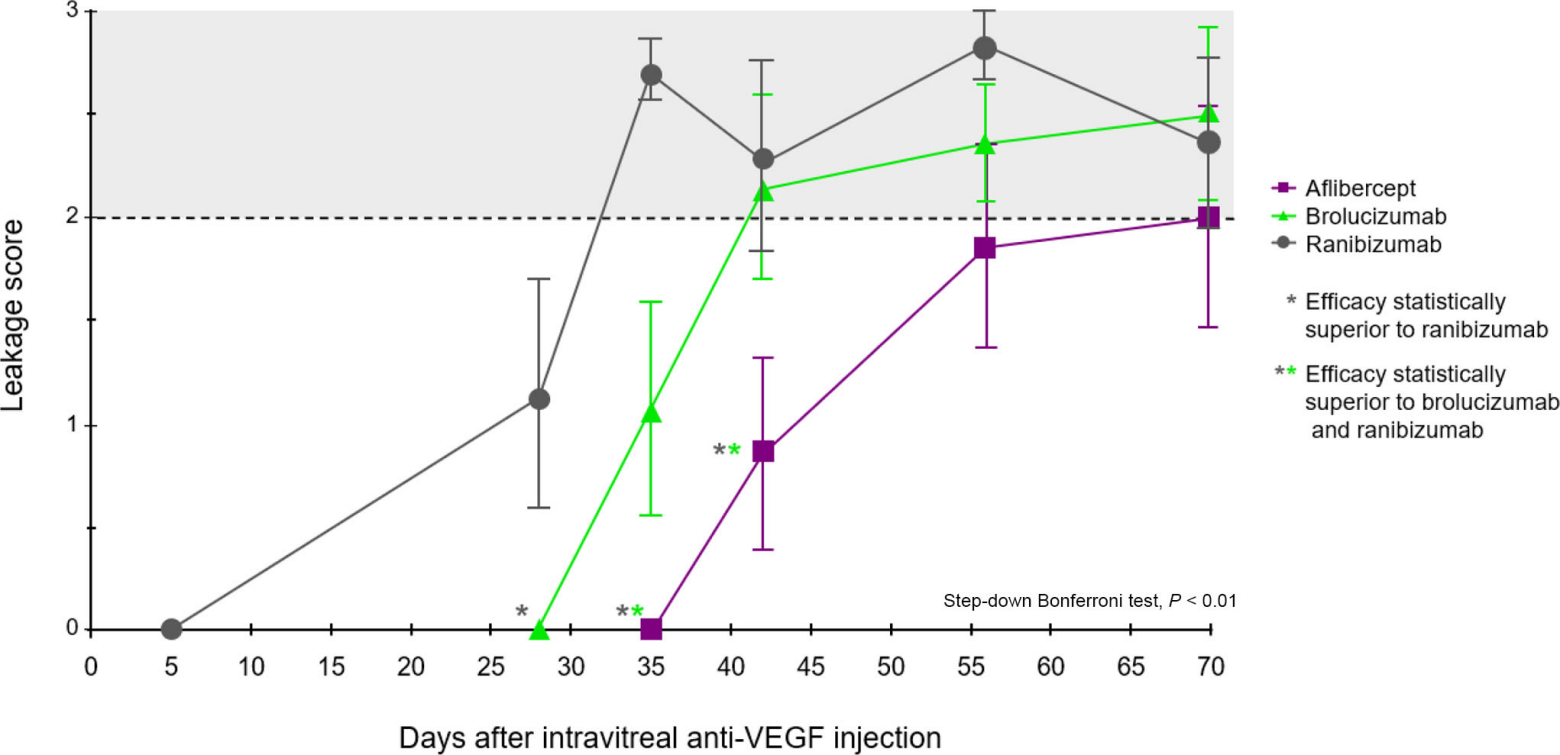
Efficacy of anti-VEGF agents to prevent VEGF-induced vascular leakage in the rabbit hyperpermeability model.

Based on the in silico model for the interaction of the different anti-VEGF agents with intravitreal injections of exogenous VEGF in rabbits, the optimized observation window of efficacy was predicted from week 3 (21 days) after intravitreal injection of each agent, through week 7 (49 days) after intravitreal injection. Within this window, all three anti-VEGF agents were predicted to lose their efficacy in preventing VEGF-induced vascular leakage in rabbits, beginning with ranibizumab, followed by brolucizumab, and then aflibercept (Supplementary Fig. S2). Figure 4 depicts the results of the in vivo Dutch belted rabbit studies, which confirm the longer efficacy of aflibercept compared with brolucizumab and ranibizumab (step-down Bonferroni test, P < 0.01). The loss of effective inhibition of retinal vascular leakage was observed at day 55 for aflibercept, day 44 for brolucizumab, and day 35 for ranibizumab.

### Pharmacokinetics and Anti-VEGF Exposure-Dependent Efficacy Evaluation in Rabbits

**Figure 5.**
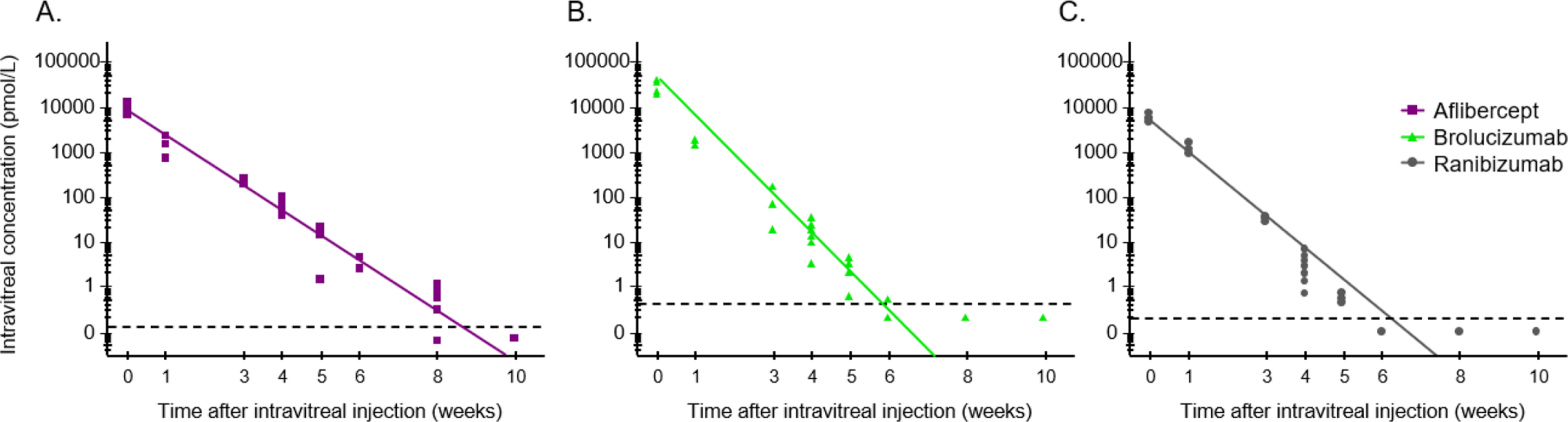
Comparison of model-predicted concentrations (pmol/mL) versus observed intravitreal exposure in vivo in rabbits of (A) aflibercept, (B) brolucizumab, and (C) ranibizumab.

Noncompartmental analysis of anti-VEGF exposure in vitreous humor indicated a two- to three-fold higher volume of distribution for brolucizumab than for aflibercept and ranibizumab. This value was larger than the typical volume of rabbit vitreous humor (1.15–1.7 mL),^20^ but still in range of typically observed values (0.72–3.14 mL).^21^ Table 1 depicts the calculated vitreous pharmacokinetic parameter estimates. The corresponding observed vitreous concentration–time profiles per agent are presented in Figure 5. The vitreal half-life of aflibercept was 5.63 days, whereas the elimination half-lives for brolucizumab and ranibizumab were similar at 3.10 and 3.15 days, respectively. Overall, the half-life predictions based on the in silico model framework closely matched the observed values.

**Table 1.** Noncompartmental Analysis of Vitreous Exposure of Aflibercept, Brolucizumab, and Ranibizumab in Dutch Belted Rabbits

Parameter	Unit	Aflibercept	Brolucizumab	Ranibizumab
Dose	µg	1000	3000	250
AUC	day·µg/mL	4140	3520	1180
C_0_	µg/mL	942	1360	287
T_last_	day	56	42	35
C_last_	µg/mL	0.07	0.02	0.03
CL	mL/day	0.24	0.85	0.21
V_ss_	mL	1.29	2.52	0.85
t_½_	days	5.63	3.15	3.10

AUC, area under the concentration–time curve; C_0_, back-extrapolated initial concentration; CL, vitreal clearance; C_last_, last observable concentration (according to T_last_); t_1/2_, half-life; T_last_, time of last measurable observed concentration; V_ss_, steady-state volume of distribution.

**Figure 6.**
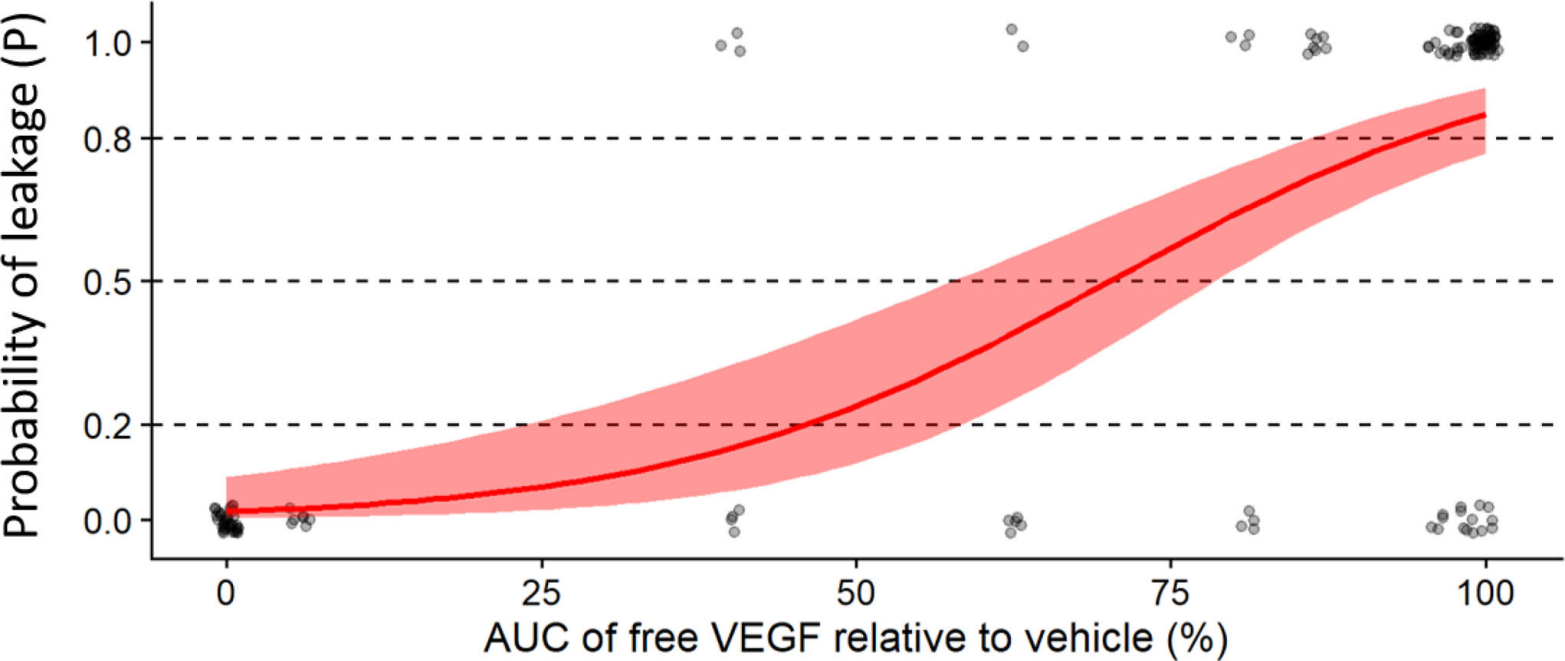
Logistic model predicting the probability of moderate-to-severe leakage based on the reduction of free exogenous VEGF in a rabbit model of VEGF-induced vascular leakage.

The post hoc comparison of the predicted pharmacokinetic profiles of the three anti-VEGF agents to the observed exposure profiles of the anti-VEGF agents is shown in Figure 6; the calculated areas under the concentration–time curve of free, that is, not blocked, exogenous injected VEGF for the three anti-VEGFs as a fraction of the maximum area under the concentration–time curve value (which would be obtained in the absence of the anti-VEGF agents), versus the probability of observing moderate to severe leakage grading in rabbits. These data can be well-described by a logistic model, as indicated by the narrow band for 95% confidence interval of the final model fit. Overall, the model performed with an accuracy of 85% (Supplementary Tables S3 and S4). Based on the model, the necessary fraction of injected VEGF that needs to be unblocked to observe moderate to severe leakage grades may be back-calculated with a certain probability. Table 2 shows these respective estimates. The probability for moderate to severe leakage increases significantly once more than approximately 40% of the injected VEGF is not captured by the respective anti-VEGF agent. This finding is consistent with our initial assessment of the data reported by Edelman et al.^19^ for this rabbit leakage model.

**Table 2.** Probability of Moderate-to-Severe Leakage versus Free VEGF in Observation Interval

Probability of Moderate-to-Severe Leakage	Free VEGF in Observation Interval (%)
0.2	46
0.5	70
0.8	94

## Discussion

This study evaluated the biochemical, cellular, and in vivo characteristics of aflibercept, brolucizumab, and ranibizumab in head-to-head studies to assess whether molecular differences translate to in vivo pharmacological differences.^9^^,^^10^ Taken together, the data consistently rank the study molecules from strongest to weakest as aflibercept, brolucizumab, and ranibizumab in binding affinity strength and calcium mobilization. The ocular half-life of brolucizumab in rabbits, as shown in Table 1, was nearly identical to the reported value of 3.0 days and the model-predicted value of 3.1 days.^22^ Schmitt^16^ reported a rabbit vitreous half-life for ranibizumab of 2.9 days, which was similar to that shown in the present study. In contrast, the predicted vitreous half-life for ranibizumab was 3.8 days, approximately 23% longer than observed. The observed vitreal half-life for aflibercept of 5.6 days was approximately 20% longer than predicted and reported values of 4.6 to 4.8 days.^16^^,^^22^^,^^23^ Li et al.^24^ examined ranibizumab, bevacizumab, and aflibercept in a similar model. Dutch belted pigmented rabbits received a single intravitreal injection of 50 μL 0.025 M of DL-alpha-aminoadipic acid to induce chronic retinal microvascular leakage, after which masked anti-VEGF agents (aflibercept 1.2 mg, ranibizumab 0.3 mg, and bevacizumab 0.75 mg) were administered intravitreally at various time points. DL-alpha-aminoadipic acid–treated eyes increased rabbit VEGF-A levels, and fluorescein angiographic images elucidated areas of prominent leakage that persisted through 48 weeks for untreated rabbit eyes. Recurrence of leakage was first shown at 6 weeks in ranibizumab-treated rabbits, followed by 8 weeks in bevacizumab and 10 weeks with aflibercept. The difference in approach for the current study was the induction of leakage with human recombinant VEGF-A, which somewhat circumvents the issue of poorer cross-reactivity of agents, such as ranibizumab or bevacizumab, to rabbits, confounding interpretation of the results. In the current study, the efficacy of ranibizumab in preventing retinal leakage was lost as early as week 5, whereas brolucizumab leakage was observed at week 6; similarly to Li et al.,^24^ aflibercept robustly prevented leakage up to week 8.

It is worth noting that these drugs are potent molecules; therefore, when cell-based studies are conducted, and depending on the individual study conditions, rank order may vary. The value of such investigations is demonstrated when trends appear to be consistent across a variety of biochemical and pharmacological parameters. The rank order of the study molecules was consistent across in vitro, in silico, and in vivo experiments, thus providing confidence that these trends may be confirmatory. The rabbit model has inherent limitations due to the lack of cross-reactivity of brolucizumab,^26^ and the weak cross-reactivity of ranibizumab to rabbit VEGF, as mentioned previously in the case of ranibizumab in the Li et al. study.^24^ This property was mitigated by using recombinant human VEGF-A165, which induces robust retinal vascular leakage in the rabbit and enables neutralization of human VEGF by all three agents. Whereas the use of recombinant human VEGF makes the model more relevant to the clinical situation regarding the molecular target, it is somewhat artificial in regard to human pathophysiology, where VEGF is released continuously, albeit in varying amounts over time.^25^ Furthermore, due to technical limitations of repeated exposure of rabbits to anesthesia, multiple cohorts were required for each time point.^11^ Multiple exposures to VEGF stimulation can also result in a potential drift of response; therefore, despite mitigating these factors by limiting cohorts to two cycles of VEGF-A exposure, opportunities for variation in the estimation of the comparative results were sizeable. Nonetheless, a clear rank order of the agents was demonstrated, which was to no small degree owing to the optimized in vivo study design based on in silico modeling. The model was refined with the pharmacokinetic data and leakage scores obtained from the anti-VEGF–treated rabbits, and can be used in the future to design similar preclinical in vivo studies for the comparison of available or future modalities using the same mode of action as the three anti-VEGF agents described herein.

## Conclusions

This study, which assessed the biochemical characteristics of aflibercept, brolucizumab, and ranibizumab using in vitro experiments, in silico modeling, and in vivo studies, explored the molecular differences of the three study molecules and whether they translated into in vivo efficacy differences in the extent and time of retinal vascular leakage. Such integrated studies evaluating in silico, in vitro, and in vivo properties characterizing the drug characteristics of anti-VEGF agents have the potential to expand our understanding of important translational properties of these agents that are currently used for the treatment of retinal diseases. Consistent with the rank orders produced from these individual characterization experiments and the reported literature, the efficacy of aflibercept in preventing retinal microvascular leakage in the rabbit in vivo model was shown to be significantly greater than brolucizumab or ranibizumab. The rank order of the results from the biochemical tests, the in vitro study, and the in vivo study remained consistent throughout.

## Supplementary Material

Supplement 1

Supplement 2

Supplement 3
