# Supplementary material for: Evaluation of Molecular Properties versus In Vivo Performance of Aflibercept, Brolucizumab, and Ranibizumab in a Retinal Vascular Hyperpermeability Model
Source: Transl Vis Sci Technol. 2022 Oct 25;11(10):36. doi: 10.1167/tvst.11.10.36 (PMC9617509; doi:10.1167/tvst.11.10.36)
Supplement: Supplement 1 [file tvst-11-10-36_s001.pdf]

Supplementary Information

PK and VEGF Binding Model

The molar concentrations of VEGF, the anti-VEGF agents, and their respective complexes were described by the following ordinary differential equations (modified from Hutton-Smith et al.).[1] The aqueous humor compartment was ignored, as no concentration data were collected for this matrix.

$$\frac{dV_{vit}}{dt} = (k_d \cdot AV_{vit} - n \cdot k_{on} \cdot V_{vit} \cdot A_{vit}) - k_{el,V} \cdot V_{vit}$$

$$\frac{dA_{vit}}{dt} = (k_d \cdot AV_{vit} - n \cdot k_{on} \cdot V_{vit} \cdot A_{vit}) + (n-1) \cdot (n \cdot k_d \cdot AVA_{vit} - k_{on} \cdot A_{vit} \cdot AV_{vit}) - k_{el,A} \cdot A_{vit}$$

$$\frac{dAV_{vit}}{dt} = -(k_d \cdot AV_{vit} - n \cdot k_{on} \cdot V_{vit} \cdot A_{vit}) + (n-1) \cdot (n \cdot k_d \cdot AVA_{vit} - k_{on} \cdot A_{vit} \cdot AV_{vit}) - k_{el,AV} \cdot AV_{vit}$$

$$\frac{dAVA_{vit}}{dt} = -(n-1) \cdot (n \cdot k_d \cdot AVA_{vit} - k_{on} \cdot A_{vit} \cdot AV_{vit}) - k_{el,AVA} \cdot AVA_{vit}$$

Capital letters indicate concentrations of individual molecules or their complexes. Subscripts are used to match binding and elimination rate constants to the respective molecules or complexes: V, VEGF; A, anti-VEGF agent; n, binding ratio of anti-VEGF agent to VEGF (1 for aflibercept, 2 for ranibizumab/brolucizumab); k_{on} and k_d, kinetic binding constants; k_{el}, elimination rate constant; $_{vit}$, vitreous humor; vol, volume of distribution.

The initial VEGF concentration at time of injection was calculated as $V_{vit,t0} = Dose_V / Vol_{vit}$. Likewise, the initial concentration of the anti-VEGF agents at the time of intravitreal injection was $A_{vit,0} = \frac{Dose_A}{Vol_{vit}}$. The initial values for the complexes were set to zero: $AV_{vit,0} = 0$ and $AVA_{vit,0} = 0$. The on-rates for VEGF binding were calculated as $k_{on} = k_d / K_D$.

Elimination rates were determined by the methodology described by Hutton-Smith et al.[1] The predicted values are summarized in **Supplementary Table S1.** The remaining intial parameters for the set-up of the pharmacokinetics model are summarized in **Supplementary Table S2**.

Supplementary Table S1. Derived vitreal elimination rates for VEGF, anti-VEGF agents, and the respective complexes.

$k_{\mathrm{el,X}}$ (1/day)	VEGF	Ranibizumab	Brolucizumab	Aflibercept
V	0.249	–	–	–
A	–	0.234	0.286	0.185
AV	–	0.191	0.210	0.165
AVA	–	0.165	0.188	–

Supplementary Table S2. Initial values for intravitreal dose, binding affinity, and interaction ratio (number of anti-VEGF agent molecules able to bind one molecule of VEGF).

	**Dose (mg)**	**Dose (nmol)**	K_D **(pmol/L)**	k_d **(1/day)**	**n (ratio)**
**Aflibercept**	1.0	10	0.45	0.14	1
**Brolucizumab**	3.0	114	1.6	0.22	2
**Ranibizumab**	0.25	5.2	100	0.18	2
**hVEGF-A165**	0.0005	0.012	—		—

Representative Images of Fluorescein Angiography Scoring

The four images shown in **Supplementary Figure S1** represent typical levels of fluorescein leakage and their corresponding fluorescein angiography score. A score of 0 represents no leakage detected. A score of 1 represents limited or low intensity of vascular leakage. A score of 2 represents significant leakage regarding both intensity of staining, but also extent of area covered by the extravascular fluorescein. This area, however, does not reach the full extent of the vascular bed comprising the optical streak. A score of 3 represents both high-intensity levels of extravascular fluorescein, as well as covering the entire vascular bed in the optical streak.

PK/PD Model for Preparation of the in vivo Study Design

In order to describe the impact of the three anti-VEGFs on the pharmacologic outcome, different simulations were run for each anti-VEGF agent with varying time of VEGF injection (typical range of 7–70 days after anti-VEGF injection). For each simulation run, the $AUC_{0\text{-}48}$ of free VEGF (V_{vit}) was calculated and compared to the AUC of free VEGF in the absence of any anti-VEGF agent.

Data from Edelman et al.[2] were used for the injection of various doses of VEGF in rabbit eyes and their subsequent impact on the observed vascular leakage for the initial PK/PD model. We planned to administer 500 ng of VEGF and read-out vascular leakage 48 h after injection in our study. Based on Edelman's evaluation, we selected the following thresholds for the prediction of anti-VEGF efficacy: reduction of free VEGF to ≥90% (equivalent to 50 ng) = full effect; reduction of free VEGF to ≤60% (equivalent to 200 ng) = no effect. This classification was applied to the simulations in order to determine the appropriate time window of investigation for each anti-VEGF agent, i.e. the time period when the VEGF blocking effect would be lost and an increase in vascular leakage would be expected. The simulated vitreal concentrations of the different anti-VEGF agents in rabbits and the predicted ability to block the effect of injected exogenous VEGF are depicted in **Supplementary Figure S2**.

Revised PK/PD model

Based on the rabbit in vivo PK and PD results, the PK/PD was refined. Binding affinity data were updated to the values reported in the main manuscript. The volume of distribution for the anti-VEGF agents was adjusted to the observed non-compartmental analysis values to accurately reflect the intravitreal concentrations during the study. As the observed half-lives matched very well with the model predictions, the elimination rates were not adjusted.

Again, for each of the anti-VEGF agents, the $AUC_{0\text{-}48}$ for free VEGF was calculated for each of the investigated time points. These values were normalized for the $AUC_{0\text{-}48}$ for free VEGF in the absence of any anti-VEGF agent. The individual animal read-outs were organised into two categories: no to low leakage (grades lower than 2), and moderate to severe leakage (grades 2 and higher). Based on this organization,

each animal was assigned to a probability of either 0 or 1 (not showing or showing moderate to severe leakage). A logistic model was used to fit the observed probability *P* values to the calculated fraction of free VEGF AUC_{frac} regardless of anti-VEGF agent:

$$P = \frac{1}{1 + \exp\left(-(\beta \cdot AUC_{frac} + \alpha)\right)}$$

Logistic regression was performed using R (version 4.1.0).[3] The obtained parameters for slope β and intercept α, as well as the confusion matrix of the model performance, are summarized in **Supplementary Table S3 and S4**.

Supplementary Table S3. Parameters estimates for the logistic pharmacodynamics model and fractions of free VEGF AUC necessary to achieve different probability levels.

Parameter	Estimate
**α**	–4.052
**β**	5.776

Supplementary Table S4. Confusion matrix depicting observed versus predicted probability of moderate-to-severe leakage

	Predicted positive	Predicted negative
**Observed positive**	85	5
**Observed negative**	19	51

REFERENCES

1. Hutton-Smith LA, Gaffney EA, Byrne HM, Maini PK, Schwab D, Mazer NA. A Mechanistic Model of the Intravitreal Pharmacokinetics of Large Molecules and the Pharmacodynamic Suppression of Ocular Vascular Endothelial Growth Factor Levels by Ranibizumab in Patients with Neovascular Age-Related Macular Degeneration. *Mol Pharm* 2016;13:2941-2950.
2. Edelman JL, Lutz D, Castro MR. Corticosteroids inhibit VEGF-induced vascular leakage in a rabbit model of blood-retinal and blood-aqueous barrier breakdown. *Exp Eye Res* 2005;80:249-258.
3. R Development Core Team. R: A Language and Environment for Statistical Computing. 2021.
