# Supplementary figures and images for: Evaluation of Molecular Properties versus In Vivo Performance of Aflibercept, Brolucizumab, and Ranibizumab in a Retinal Vascular Hyperpermeability Model
Source: Transl Vis Sci Technol. 2022 Oct 25;11(10):36. doi: 10.1167/tvst.11.10.36 (PMC9617509; doi:10.1167/tvst.11.10.36)

**Supplementary Figure S1.** Representative images of fluorescein angiography scoring.

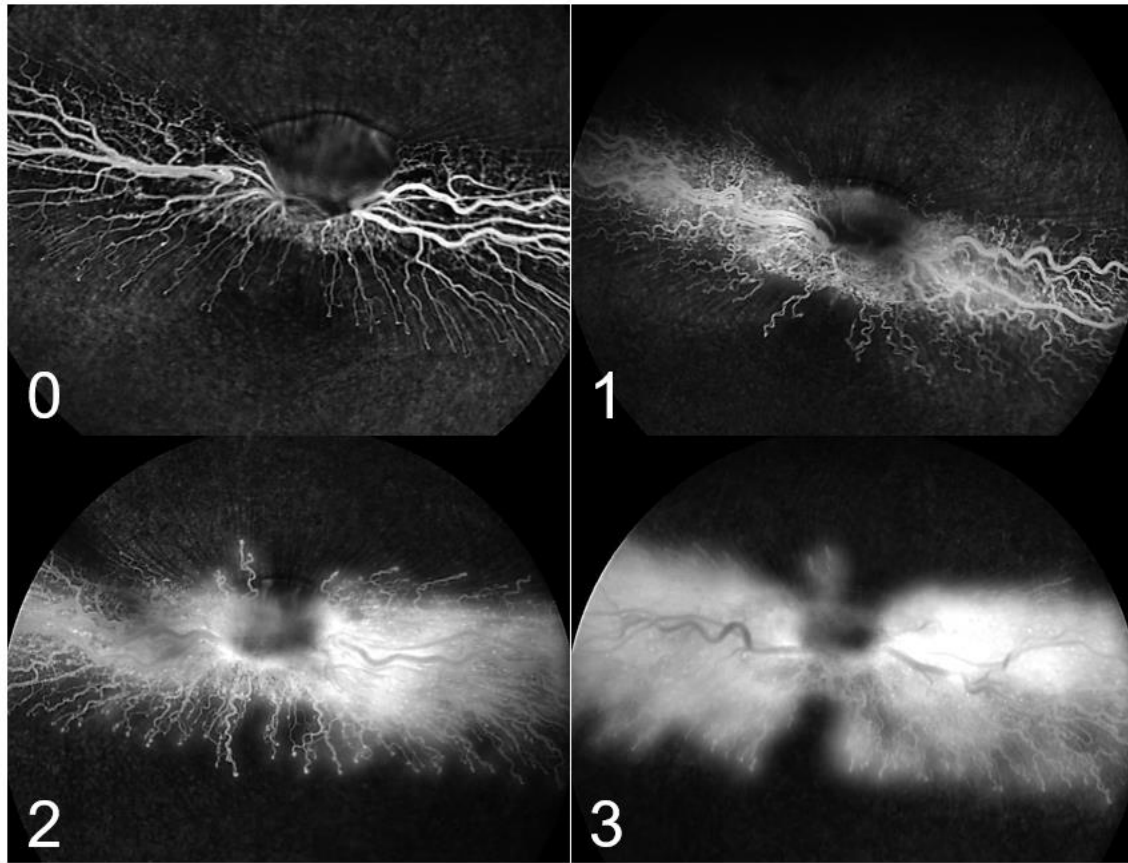

Supplement: Supplement 2 [file tvst-11-10-36_s002.pdf]
