# Supplementary material for: Evaluation of Molecular Properties versus In Vivo Performance of Aflibercept, Brolucizumab, and Ranibizumab in a Retinal Vascular Hyperpermeability Model
Source: Transl Vis Sci Technol. 2022 Oct 25;11(10):36. doi: 10.1167/tvst.11.10.36 (PMC9617509; doi:10.1167/tvst.11.10.36)

Supplementary Figure S2. Simulated vitreous drug concentration–time profiles in rabbits (**A**) and predicted time period when the VEGF blocking effect would be lost and an increase in induced vascular leakage would be expected (**B**).

A.

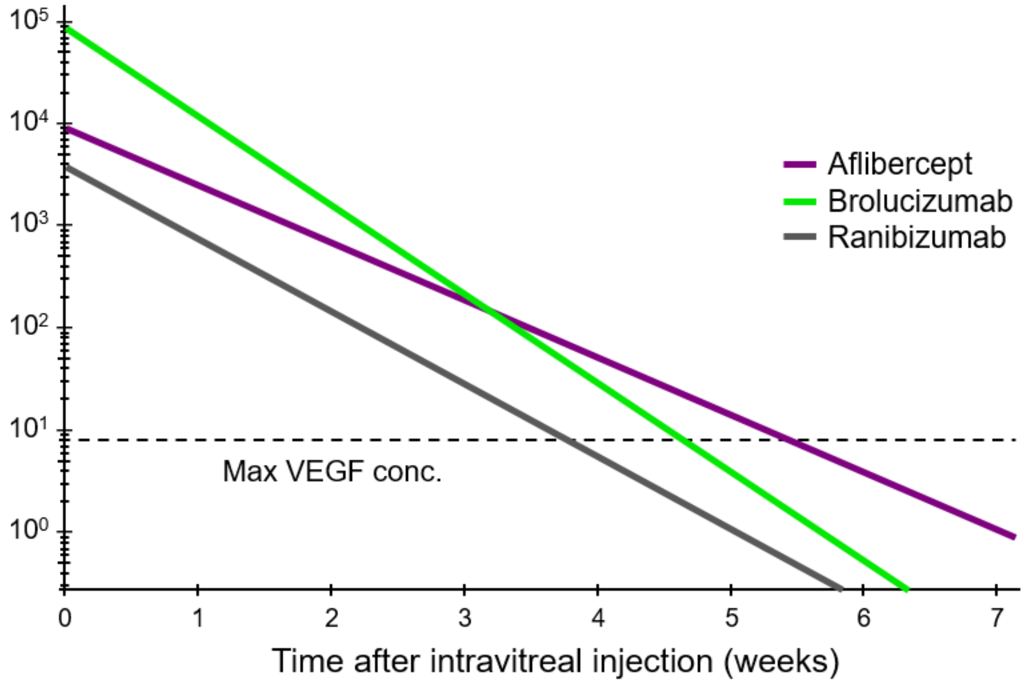

B.

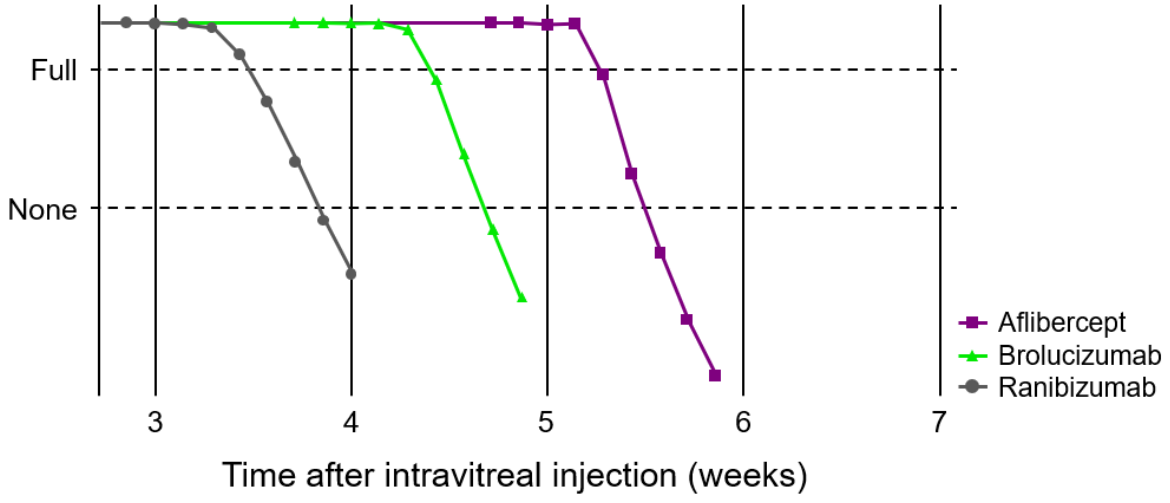

Supplement: Supplement 3 [file tvst-11-10-36_s003.pdf]
